# Изменение кишечного микробиома пациентов с язвенным колитом после трансплантации кишечной микробиоты

**DOI:** 10.18699/VJ20.610

**Published:** 2020-03

**Authors:** А.Ю. Тикунов, В.В. Морозов, А.Н. Швалов, А.В. Бардашева, Е.В. Шрайнер, О.А. Максимова, И.О. Волошина, В.В. Морозова, В.В. Власов, Н.В. Тикунова

**Affiliations:** Институт химической биологии и фундаментальной медицины Сибирского отделения Российской академии наук, Новосибирск, Россия; Институт химической биологии и фундаментальной медицины Сибирского отделения Российской академии наук, Новосибирск, Россия; Государственный научный центр вирусологии и биотехнологии «Вектор» Роспотребнадзора Российской Федерации, р. п. Кольцово, Новосибирская область, Россия 3 ООО «Центр персонализированной; Институт химической биологии и фундаментальной медицины Сибирского отделения Российской академии наук, Новосибирск, Россия; Институт химической биологии и фундаментальной медицины Сибирского отделения Российской академии наук, Новосибирск, Россия; ООО «Центр персонализированной медицины», Новосибирск, Россия; ООО «Центр персонализированной медицины», Новосибирск, Россия; Институт химической биологии и фундаментальной медицины Сибирского отделения Российской академии наук, Новосибирск, Россия; Институт химической биологии и фундаментальной медицины Сибирского отделения Российской академии наук, Новосибирск, Россия; Институт химической биологии и фундаментальной медицины Сибирского отделения Российской академии наук, Новосибирск, Россия

**Keywords:** microbiome, ulcerative colitis, 16S rRNA profiling, fecal microbiota transplantation, микробиом, язвенный колит, 16S рРНК профилирование, трансплантация кишечной микробиоты

## Abstract

Микробиота кишечника человека – это динамическая система, находящаяся под воздействием организма хозяина и внешних факторов. Возникающие нарушения кишечной микробиоты могут привести к патологическим состояниям, включая воспалительные и онкологические заболевания желудочно-кишечного тракта. Одним из возможных способов воздействия на микробиоту кишечника является фекотрансплантация (ФТ) – введение кишечной микробиоты от здорового донора в кишечный тракт пациента. В настоящее время в ряде стран этот метод используется для нормализации микробиоты кишечника, в основном при хронических воспалительных заболеваниях кишечника. В России (Новосибирск) уже несколько лет ведутся пилотные исследования эффективности ФТ при язвенном колите. Цель данной работы – оценить изменение микробиома кишечника 20 пациентов с язвенным колитом после однократного проведения ФТ. Основной метод – сравнительный анализ библиотек последовательностей 16S рибосомальной РНК, созданных на основе образцов, полученных от пациентов с язвенным колитом до и после ФТ и секвенированных на платформе Illumina MiSeq. Результаты исследования показали, что ФТ привела к увеличению среднего биоразнообразия последовательностей в образцах, полученных после ФТ, по сравнению с образцами, собранными до ФТ, хотя разница не была статистически достоверной. Доля последовательностей Firmicutes, являющихся доминирующей компонентой кишечной микробиоты здоровых людей, уменьшилась (~32 % vs. >70 %), а доля последова- тельностей Proteobacteria увеличилась (>9 % vs. <5 %). В некоторых образцах, собранных до ФТ, были обнаружены последовательности патогенных представителей Firmicutes и Proteobacteria, включая Acinetobacter spp., Enterococcus spp., Klebsiella pneumoniae, Proteus mirabilis, Staphylococcus aureus, Stenotrophomonas maltophylia, Streptococcus spp. В большинстве случаев после ФТ доля таких последовательностей резко сократилась. Исключение составили последовательности Clostridium difficile, содержание которых в образцах почти половины пациентов составляло менее 0.5 %; после ФТ доля последовательностей C. difficile значительно уменьшилась лишь у трех пациентов. Следует отметить, что после ФТ повысилось на порядок содержание Lactobacillus spp. и существенно расширился их видовой состав. По результатам исследования можно сделать предварительное заключение о том, что даже однократная процедура ФТ приводит к повышению биоразнообразия микробиоты пациентов и оптимизации ее таксономического состава.

## Abstract (en)

Intestinal human microbiota is a dynamic system that is under the pressures of its host organism and external factors. Microbiota disruption caused by these factors can lead to severe diseases including inflammatory and oncological diseases of the gastrointestinal tract. One of the possible approaches in managing the intestinal microbiota is fecal microbiota transplantation (FT) – transfer of the microbiota from the stool of a healthy donor to the intestinal tract of a recipient patient. Currently, this procedure is recognized as an efficacious method to normalize the intestinal microbiota mainly in inflammatory diseases of the gastrointestinal tract. In Russia, pilot studies of the effectiveness of FT in patients with ulcerative colitis have been conducted for several years, and these studies were started in Novosibirsk. The aim of this study was to assess the change of intestinal microbiome in 20 patients with ulcerative colitis after a single FT procedure. The main method is a comparative analysis of 16S ribosomal RNA sequence libraries constructed using fecal samples obtained from patients with ulcerative colitis before and after FT and sequenced on the Illumina MiSeq platform. The obtained results showed that FT led to an increase in average biodiversity in samples after FT compared to samples before FT; however, the difference was not significant. In the samples studied, the proportion of Firmicutes sequences, the major gastrointestinal microbiota of healthy people, was decreased (~32 % vs. >70 %), while the proportion of Proteobacteria sequences was increased (>9 % vs. <5 %). In some samples collected before FT, sequences of pathogenic Firmicutes and Proteobacteria were detected, including Acinetobacter spp., Enterococcus spp., Klebsiella pneumoniae, Proteus mirabilis, Staphylococcus aureus, Stenotrophomonas maltophylia, Streptococcus spp. In most cases, the proportion of such sequences after FT substantially decreased in appropriate samples. The exception was the Clostridium difficile sequences, which accounted for <0.5 % of the sequences in samples from almost half of the patients and after FT, the share of such C. difficile sequences was significantly reduced only in samples from three patients. It should be noted that the proportion of Lactobacillus spp. increased ten-fold and their species composition significantly expanded. According to the obtained results, a preliminary conclusion can be made that even a single FT procedure can lead to an increase in the biodiversity of the gastrointestinal microbiota in patients and to the optimization of the taxonomic composition of the microbiota.

## Введение

Широкое применение технологий NGS (next generation sequencing) обеспечило детальную характеризацию микробных сообществ, преимущественно бактериальных, ассоциированных с организмом человека. В настоящее время микробиота кишечника человека рассматривается как динамическая система, находящаяся под воздействием организма хозяина и внешних факторов (Fujimura et al., 2010; Qin et al., 2010). Известно, что основными компонентами нормальной кишечной микробиоты человека являются представители Firmicutes и Bacteroidetes, хотя коровая группа конкретных видов бактерий не совпадает у разных здоровых индивидуумов (Donaldson et al., 2016), и различные по составу варианты нормальной микробиоты способны обеспечить стабильное функционирование этого сложного микробного сообщества (Lozupone et al., 2012).

Дисбаланс кишечной микробиоты, возникающий под действием внешних или внутренних факторов, может привести к патологическим состояниям, включая не только воспалительные и онкологические заболевания желудочно-кишечного тракта, но и нарушения иммунной системы, диабет второго типа, сосудистые заболевания и даже нарушения функций головного мозга (O’Hara, Shanahan, 2006). Одним из возможных способов воздействия на микробиоту кишечника, наряду с использованием антибиотиков, пробиотиков и пребиотиков, является фекотрансплантация (ФТ) – введение кишечной микробиоты от здорового донора в кишечный тракт пациента. Считается, что до нашей эры применение ФТ практиковали в Китае, а в наше время эту процедуру впервые провели в 1958 г. при лечении пациента с энтероколитом (Eiseman et al., 1958). Однако лишь недавно метод стал успешно использоваться для нормализации микробиоты кишечника при различных заболеваниях, включая хронические воспалительные заболевания кишечника (Aas et al., 2003; Khoruts et al., 2010; Angelberger et al., 2013; Pigneur, Sokol, 2016; Vaughn et al., 2016; Kang et al., 2017; Paramsothy et al., 2017; Staley et al., 2017).

Наибольшая результативность применения ФТ показана при псевдомембранозном колите, ассоциированном с Clostridium (Clostridioides) difficile (Drekonja et al., 2015; Khoruts, Sadowsky, 2016; Cheng, Fisher, 2017; Staley et al., 2017; Goldenberg et al., 2018). Быстрое улучшение состояния пациентов и высокая эффективность ФТ (более 80 %) при этих инфекциях была подтверждена в многоцентровых рандомизированных клинических испытаниях (van Nood et al., 2013; Cammarota et al., 2015), и сейчас в США и в европейских странах рекомендуется применять ФТ уже после второго или третьего эпизода C. difficile-ассоциированного колита (Debast et al., 2014).

Имеются свидетельства того, что ФТ может быть полезной и при язвенном колите (ЯК). Эффективность применения ФТ для лечения пациентов с ЯК в различных исследованиях существенно варьировала – от 20 до 92 % (Angelberger et al., 2013; Kellermayer et al., 2015). В рандомизированных клинических испытаниях ремиссия зарегистрирована на уровне 25–27 %, что, впрочем, статистически значимо превышало эффект плацебо (Moayyedi et al., 2015; Rossen et al., 2015; Paramosthy et al., 2017). Патогенез ЯК не вполне ясен; полагают, что он имеет сложную природу и опосредован нарушениями кишечной микробиоты, генетической предрасположенностью и экологическими факторами (Shen et al., 2018). Как и при C. difficile-ассоциированном колите, биоразнообразие микробиоты кишечника при ЯК существенно снижено, уменьшено количество представителей Bacteroidetes и Firmicutes. При этом в микробиоте пациентов с ЯК увеличивается количество представителей Proteobacteria и Actinomycetes, выявляются C. difficile, Helicobacter pylori, Salmonella spp., Yersinia spp. и энтероинвазивные Escherichia coli (Okhusa et al., 2003; Saebo et al., 2005; Gradel et al., 2009; Sonnenberg, Genta, 2012; Deshpande et al., 2013; Reddy, Brandt, 2013; Shen et al., 2018). До сих пор не ясно, является ли ЯК результатом нарушенного иммунного ответа на нормальную микробиоту, или это нормальный иммунный ответ на дисбаланс в микрофлоре кишечника (Cheng, Fisher, 2017).

Несмотря на кажущуюся очевидность, механизмы положительного действия ФТ при ЯК не вполне ясны. Полагают, что эффективность ФТ связана с увеличением разнообразия микробиоты кишечника, что приводит к повышению обилия «полезных» бактерий и препятствует колонизации кишечника патогенными бактериями (Broecker et al., 2016; Chehoud et al., 2016; Khoruts, Sadowsky, 2016). Однако неизвестно, вовлечены ли в этот процесс другие механизмы, включая возможное влияние виробиоты, действие иммунной системы пациента, привнесение регуляторных высоко- и низкомолекулярных соединений при ФТ; также до сих пор не определен список видов бактерий, обусловливающих нормализацию микробиоты. Цель данного исследования – оценить изменение микробиома кишечника пациентов с ЯК после проведения ФТ на основе профилирования 16S рибосомальной РНК в образцах, полученных до и после лечения.

## Материалы и методы

В работе использовали образцы фекалий, полученные от 20 пациентов (27–57 лет) с диагнозом ЯК. Диагноз подтверждали на основании результатов изучения уровня фекального кальпротектина, данных фиброколоноскопии и гистологического исследования биоптатов, взятых из разных отделов толстой и подвздошной кишок. Образцы пациентов, в которых рутинными методами были обнаружены C. difficile, в исследование не вовлекались. Все пациенты предоставили информированное согласие с проводимым исследованием и анонимной обработкой данных. Образцы собирали за один-два дня до ФТ и через 7–12 дней после ФТ. Донорами были молодые здоровые добровольцы (20–39 лет) без хронических заболеваний, не перенесшие инфекции и не подвергавшиеся госпитализации по крайней мере последние два месяца. Все доноры прошли обследование, включающее в себя общий и биохимический анализы крови, а также ИФА крови на наличие лямблий, токсокар, описторхов, аскарид, трихинелл. Кроме того, с использованием стандартных тест- систем подтверждали отсутствие у доноров возбудителя сифилиса, ВИЧ-1 и ВИЧ-2, вирусов гепатита В и С. Также рутинными методами проводили анализ фекалий на дисбиоз и на отсутствие патогенной микрофлоры (C. difficile, Campilobacter jejuni, Salmonella spp., Shigella spp., энтероинвазивная Escherichia coli, Cryptosporidium spp., Cyclospora spp., Giardia spp., Isospora spp.), ротавирусов А, норовирусов I и II и аденовирусов F, а также гельминтов и их яиц. Пилотное исследование было одобрено Локальным этическим комитетом Автономной некоммерческой организации «Центр новых медицинских технологий в Академгородке».

По 50 мг каждого образца от пациентов суспендировали в 300 мкл 0.9 % NaCl и центрифугировали при 2 тыс. об. в течение 10 мин. Суммарную ДНК очищали из 100 мкл осветленной клеточной суспензии с помощью набора для выделения ДНК из клеток тканей и крови («БиоЛабМикс », Россия) с добавлением лизоцима для повышения эффективности извлечения ДНК из грамположительных бактерий. С использованием полученной ДНК в качестве матрицы, фьюжн-праймеров (NEB-FF 5ʹ-ACACTCTTTC CCTACACGACGCTCTTCCGATCTCTACGGGAGGCA GCAG-3ʹ, NEB-FR 5ʹ-GTGACTGGAGTTCAGACGTGT GCTCTTCCGATCTGGACTACCGGGGTATCT-3ʹ) и высокоточной полимеразы Q5 (New England Biolabs, США) проводили амплификации фрагмента гена 16S рРНК, содержащего вариабельные участки V3–V4. Продукты амплификации очищали электрофоретически в геле из легкоплавкой SeaKem GTG-агарозы (Lonza, США). Обогащение полученных ампликонов, введение баркодов и служебных последовательностей для дальнейшего секвенирования на платформе MiSeq выполняли, используя полимеразу Q5 и набор олигонуклеотидов Dual index set (New England Biolabs), согласно инструкции производителя. Полученные библиотеки очищали на магнитных частицах AMPure XP (Beckman Coulter, США); концентрацию ДНК измеряли с помощью набора Qubit dsDNA HS (Life Technologies, США). По результатам измерений библиотеки объединяли в пул таким образом, чтобы соотношение ДНК библиотек в пуле было приблизительно равным. Секвенировали на высокопроизводительном секвенаторе MiSeq с набором реагентов MiSeq reagent kit v2 2 × 250-cycles (Illumina, США).

Результаты секвенирования анализировали с использованием пакета программного обеспечения UGENE v.1.32 (Unipro UGENE, Россия). Полученные риды картировали на базу данных 16S рРНК, размещенную на Национальном сервере NSBI (США), с использованием пакета Clark. Предварительно из последовательностей ридов удаляли последовательности адаптеров и проводили фильтрацию ридов по качеству. Риды анализировали двумя методами: с помощью генерации операционных таксономических единиц (OTU) с последующим картированием последовательностей на полученные OTU в пакете программ Usearch-9.2 и путем классификации ридов алгоритмом Kraken по базе данных известных последовательностей 16S рРНК Silva v.132 (full). В первом случае OTU генерировали алгоритмом unoise2 с отбраковкой химерных последовательностей и учетом ошибок чтения. Таблицы полученных частот встречаемости OTU были обработаны в среде R3.3.3. Во втором случае риды картировали на базу данных 16S рРНК Silva с помощью алгоритма seedkraken с использованием разреженного k-мера со специальной решеткой, позволяющей увеличить специфичность классификации. Индекс Шеннона рассчитывали в пакете программ R; достоверность различий между индексами Шеннона определяли с помощью t-теста Хатчесона. Визуализацию результатов анализа библиотек последовательностей методом главных координат PCoA проводили на основе матриц дистанций с использованием пакета программ vegan.

## Результаты и обсуждение

На основе ДНК, выделенной из образцов фекалий от 20 пациентов с ЯК до и после ФТ, было сконструировано 40 библиотек фрагментов гена 16S рРНК. Фрагменты гена включали вариабельные участки V3 и V4, используемые обычно для таксономической классификации бактерий (Chakravorty et al., 2007; Wang, Qian, 2009). В результате секвенирования библиотек, которые были созданы на основе образцов, собранных до ФТ, получено от 106 411 до 1 751 663 ридов (в среднем 141 010). Библиотеки из образцов, собранных после ФТ, содержали от 107 042 до 173 855 ридов (в среднем 142 060). По результатам классификации в библиотеках из первой и второй групп к определенному типу прокариот были таксономически отнесены в среднем 99.7 и 99.6 % ридов соответственно. Лишь незначительная часть последовательностей осталась неклассифицированной. Всего выявлены последовательности 13 типов бактерий, основными из которых были Firmicutes, Bacteroidetes и Proteobacteria. В трех образцах выявлены последовательности архей, принадлежащих к роду Methanobrevibacter (тип Euryarchaeota), однако доля таких последовательностей в соответствующих библиотеках не превышала 0.1 %.

Анализ полученных данных показал, что биоразнообразие бактериальных сообществ в образцах от пациентов до и после ФТ различается. Так, индекс Шеннона для выборки образцов от пациентов после лечения (3.43 ± 0.71) был выше, чем для образцов, взятых до лечения (3.05 ± 0.67), хотя разница статистически недостоверна (рис. 1). Однако при попарном сравнении индексов Шеннона для образцов, полученных от одного пациента до и после ФТ, различия были во всех случаях статистически достоверными (SD, 0.011–0.019); при этом у 15 пациентов биоразнообразие микробных сообществ значимо увеличилось и лишь у 5 пациентов – уменьшилось.

**Fig. 1.**
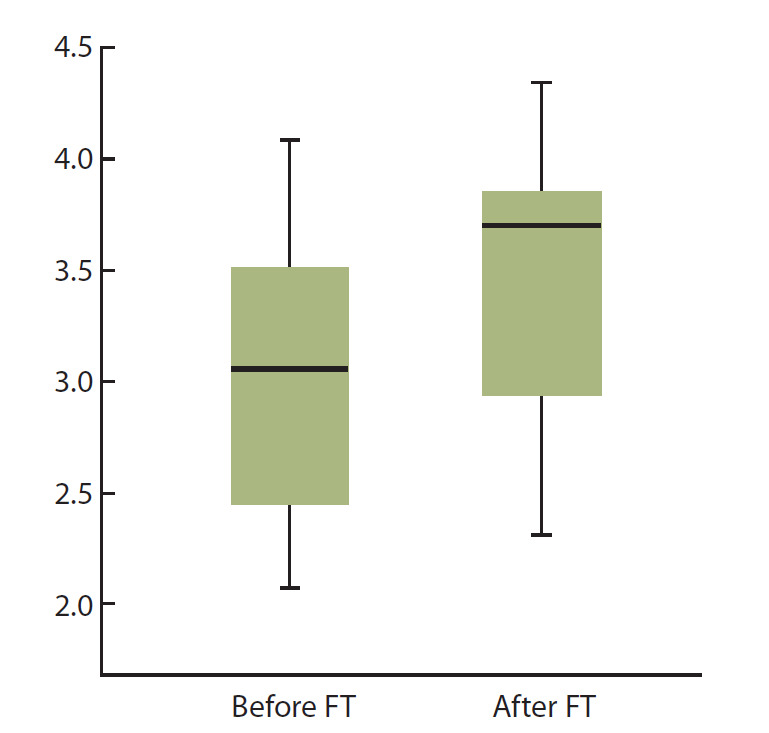
Shannon index, reflecting the biodiversity of sequences in samples obtained from patients before and after FT.

Большее биоразнообразие сообществ в образцах, полученных после ФТ, подтвердилось при анализе данных методом главных координат (рис. 2). Видно, что 60 % образцов, собранных до лечения, находятся в области отрицательных значений первой главной координаты, тогда как 65 % образцов, взятых после лечения, находятся в области положительных значений. Аналогично в области положительных значений второй главной координаты находится лишь 45 % образцов, полученных до лечения, и 70 % образцов после ФТ. Отметим, что в образцах от 15 пациентов (75 %) после проведения ФТ значение увеличилось в области и первой, и второй главных координат; еще у четырех пациентов значение увеличилось в области хотя бы одной из главных координат; только для пары образцов от одного пациента (№ 18) биоразнообразие уменьшилось после ФТ в области значений обеих главных координат (см. рис. 2). Тем не менее при уменьшении значений в области хотя бы одной главной координаты после ФТ индекс Шеннона для этой же пары образцов статистически значимо уменьшался.

**Fig. 2.**
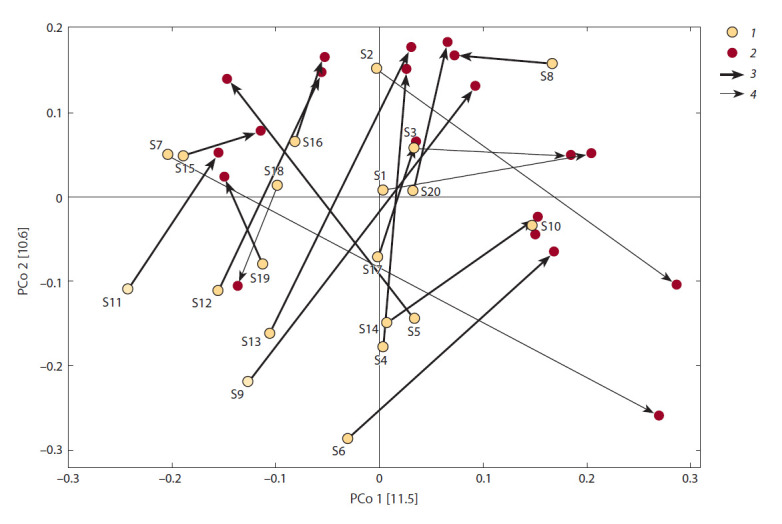
Visualization of sequence library analysis by the method of principal coordinates PCoA made on matrices of distances: 1, data for libraries from samples obtained before FT; 2, from samples obtained after FT. 3, 4, lines connecting two samples from one patient for which biodiversity (3) increases or (4) decreases after FT. Designations S1–S20 correspond to sample numbers before FT. The values of the first and second principal coordinates are presented on the OX and OY axes, respectively.

Полученные данные подтверждают возможность повышения биоразнообразия бактериального сообщества кишечника после проведения ФТ. Следует подчеркнуть, что в настоящем исследовании мы сравнивали исходный микробиом пациентов и микробиом после первой процедуры ФТ. Согласно опубликованным сведениям зарубежных авторов, даже после нескольких процедур ФТ статистически значимое повышение биоразнообразия микробиоты регистрируется не у всех пациентов (Angelberger et al., 2013).

Анализ таксономического состава показал, что в образцах до и после ФТ в среднем 55.7 % (от 12.7 до 72.8 %) и 48.3 % (от 15.9 до 92.3 %) выявленных последовательностей соответственно принадлежали к типу Firmicutes (рис. 3), 32.3 % (от 4.4 до 64.4 %) и 33.7 % (от 1.7 до 60.7 %) – к типу Bacteroidetes, а 9.2 % (от 0.4 до 81.3 %) и 6.2 % (от 0.7 до 31.5 %) – к типу Proteobacteria. Четвертыми по встречаемости были последовательности Fusobacteria, в среднем 3.4 % по всем библиотекам, однако их доля значительно увеличилась после ФТ: от <0.1 (0–0.3 %) до 6.7 % (0.1–9.4 %). После ФТ увеличилась также доля последовательностей Actinobacteria, хотя и не так значительно – от 1.9 (0.1–10.7 %) до 3.1 % (0.2–19.4 %). Последовательности Verrucomicrobia присутствовали в каждом образце в небольших количествах, а их встречаемость после ФТ уменьшилась в среднем с 0.4 до 0.2 %. Остальные последовательности встречались лишь в отдельных образцах, не превышая 0.1 % от всех последовательностей в этом образце.

Известно, что микробиота здоровых людей состоит из постоянных и транзиторных видов, относящихся более чем к 17 типам, включая Firmicutes (>70 %), Bacteroidetes (>30 %), протеобактерии (<5 %), актинобактерии (<2 %), Fusobacteria и Verrucomicrobia (<1 %) (Belizário et al., 2018). Полученные нами результаты коррелируют с данными других исследователей, свидетельствующими о пониженном биоразнообразии микробиоты кишечника при ЯК (Manichanh et al., 2012; Machiels et al., 2014; Bajer et al., 2017). Так, в исследуемых образцах от пациентов в среднем присутствовало существенно меньше последовательностей Firmicutes, что хорошо согласуется с на-блюдениями других авторов (Machiels et al., 2014). В основном Firmicutes были представлены последовательностями классов Clostridia (рис. 4), в среднем 47.4 и 40.6 % в библиотеках из образцов, полученных до и после ФТ соответственно. Из них доминировали Faecalibacterium prausnitzii (15.7 и 11.3 % соответственно) и Roseburia hominis (2.3 и 0.5 %), ответственные за расщепление широкого спектра углеводов, включая крахмал и инулин, с образованием бутиратов (Duncan et al., 2007; Machiels et al., 2014). Также были представлены последовательности классов Negativicutes (6.8 и 4.0 %) и Bacilli (1.3 и 2.8 %). Следует отметить, что, несмотря на относительно невысокую встречаемость Bacillus spp. и Lactobacillus spp., их доля после ФТ повысилась в 3.5 и 11 раз соответственно. При этом существенно расширился видовой состав лактобацилл, которые, как известно, не только участвуют в расщеплении лактозы и других углеводов, но и являются антагонистами по отношению к патогенным микроорганизмам, вытесняя их из микробного сообщества кишечника человека.

Доля последовательностей, принадлежащих к типу Bacteroidetes, в исследуемых образцах от пациентов с ЯК не была снижена (см. рис. 3 и 4) и составила около трети от всех последовательностей, что отличается от наблюдений других исследователей (Machiels et al., 2014). В основном этот тип был представлен последовательностями Bacteroides spp. и Prevotella spp., причем после ФТ доля последовательностей бактероидов уменьшилась в среднем с 19.7 до 9.6 %, а последовательностей, принадлежащих к роду Prevotella, существенно увеличилась, с 3.7 до 14.6 %. Известно, что большинство бактероидов, обитающих в кишечнике человека, способны разлагать разнообразные растительные полисахариды (Flint et al., 2012), причем в кишечной микробиоте жителей западных стран преобладают Bacteroides spp., а в микробиоте населения из стран с преимущественно растительной диетой – Prevotella spp. (Ley, 2016).

**Fig. 3.**
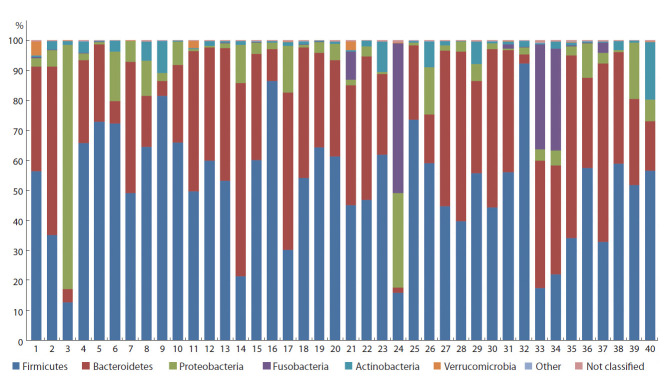
Taxonomic classification of OTUs at the phylum level based on the Silva v.132 (full) database. Here and in Fig. 4 samples 1–20 were obtained from patients before FT (enumeration follows Fig. 2); samples 21–40 were collected after FT. Samples 1 and 21, 2 and 22, etc. were obtained from the first, second, etc. patients, respectively. Phyla with representation exceeding 0.1 % are shown.

**Fig. 4.**
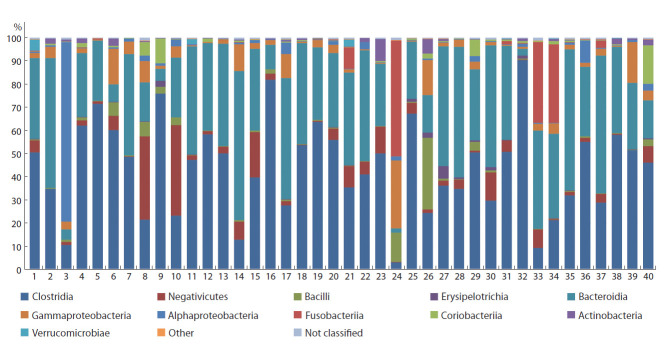
Taxonomic classification of OTUs at the class level based on the Silva v.132 (full) database. Classes with representation exceeding 0.1 % are shown.

Отметим, что доля последовательностей Proteobacteria (см. рис. 3) в образцах, собранных до ФТ (в среднем 9.2 %), превышала таковую, обычно регистрируемую у здоровых людей (<5 %) (Belizário et al., 2018). Это объясняется повышенным содержанием Salmonella spp. в микробных сообществах пациентов с ЯК и наличием в отдельных образцах в большом количестве последовательностей патогенных бактерий, способных вызвать желудочно- кишечные заболевания (более 9 % Acinetobacter spp., 0.5–1 % Klebsiella pneumoniae), что установлено и в других исследованиях (Gradel et al., 2009; Shen et al., 2018). В единичных образцах были обнаружены также Proteus mirabilis и Stenotrophomonas maltophilia, доля которых не превышала 0.1 %. Надо сказать, что после проведения ФТ содержание перечисленных последовательностей патогенных Proteobacteria в образцах существенно уменьшилось, порой до 0.02 % и менее.

Кроме последовательностей патогенных Proteobacteria, в некоторых образцах, полученных от пациентов до ФТ, были обнаружены последовательности патогенных представителей Firmicutes. Так, в девяти образцах найдены последовательности C. difficile, доля которых превышала 0.5 %. Повышенная встречаемость C. difficile в кишечной микробиоте пациентов с ЯК отмечалась и ранее (Deshpande et al., 2013; Reddy, Brandt, 2013). Помимо последовательностей C. difficile, в некоторых образцах обнаружены последовательности Staphylococcus aureus (0.1–0.9 %), Streptococcus spp. и Enterococcus spp. (~0.1 %). Как и в случае с патогенными Proteobacteria, доля этих последовательностей после ФТ в соответствующих образцах резко уменьшилась. Исключение составили образцы с C. difficile: доля их последовательностей уменьшилась после ФТ только в трех образцах из девяти, составив <0.2 %.

## Заключение

Таким образом, исследованы биоразнообразие и таксономический состав последовательностей фрагмента гена 16S рРНК, ассоциированных с кишечной микробиотой у 20 пациентов с ЯК до и после ФТ. Результаты показали, что однократное проведение процедуры ФТ привело к увеличению среднего биоразнообразия последовательностей в образцах, полученных после ФТ, по сравнению с образцами, собранными до ФТ, хотя разница не была статистически достоверной. Доля последовательностей Firmicutes, являющихся доминирующей компонентой кишечной микробиоты здоровых людей, была снижена (~32 % vs. >70 %), а доля последовательностей Proteobacteria увеличена (>9 % vs. <5 %). В некоторых образцах, собранных до ФТ, обнаружены значимые содержания последовательностей патогенных представителей Firmi-cutes и Proteobacteria, включая Acinetobacter spp., Enterococcus spp., K. pneumoniae, P. mirabilis, S. aureus, St. maltophilia, Streptococcus spp. В большинстве случаев после однократной процедуры ФТ доля таких последовательностей резко сократилась. Исключение составили последовательности C. difficile, которые были обнаружены (около 0.5 % и выше) в образцах почти половины пациентов с ЯК; после ФТ доля последовательностей C. difficile значительно уменьшилась лишь у трех пациентов. Следует отметить, что после ФТ содержание Lactobacillus spp. повысилось на порядок и существенно расширился видовой состав лактобацилл.

Результаты исследования позволяют сделать предварительный вывод, что даже однократная процедура ФТ может привести к повышению биоразнообразия микробиоты и оптимизации ее таксономического состава. Однако для того чтобы сделать заключение об эффективности такого лечения, длительности ремиссии и стабильности изменений микробиоты кишечника у пациентов с ЯК, требуются дальнейшие наблюдения за этими пациентами и анализ кишечного микробиома после последующих процедур ФТ.

## Conflict of interest

The authors declare no conflict of interest.
